# Structural and Functional Characterization of SOSTDC1

**DOI:** 10.1063/4.0001017

**Published:** 2025-10-27

**Authors:** Melissa M Gouge, Gregory Gipson, Chandramohan Kattamuri, Thomas B. Thompson

**Affiliations:** University of Cincinnati, Cincinnati, Ohio, USA

## Abstract

The differential screening-selected gene in neuroblastoma (DAN) family of antagonists are secreted extracellular proteins which preferentially bind to and inhibit BMP ligands. SOSTDC1 (Sclerostin domain containing protein 1) is a divergent member of the DAN family which has previously been shown to perform dual roles as a BMP inhibitor and a Wnt modulator. With the current understanding of SOSTDC1, it is challenging to determine the effect of SOSTDC1 activity within the BMP-Wnt signaling gradients, which are commonly seen in development, gut homeostasis, and female reproduction. SOST, the closest homologue of SOSTDC1, is a monomeric inhibitor of WNT signaling, binding to LRP6 β-propeller domain 1 through a conserved motif (NXI) but does not inhibit BMPs. Our lab has previously shown that unlike SOST, SOSTDC1 exists as a stable non-disulfide linked dimer, which binds to and inhibits BMPs. In this study we aimed to characterize the dimer conformation of SOSTDC1, focusing on how differences between SOST and SOSTDC1, particularly the dimeric structure, are responsible for their differences in function. We determined an X-ray crystal structure of a truncated SOSTDC1 dimer at 2.1Å resolution. The crystal structure, which was also validated by SAXS, revealed a divergent dimer interface when compared to other DAN family members. This conformation allows the NXI motif to be solvent exposed, leaving two NXI motifs free for LRP6 binding. *In vitro* cell signaling assays showed that SOSTDC1 inhibits Wnt1 (LRP6 β- propeller domains 1-2) mediated signaling. Surprisingly, when tested against Wnt3a (LRP6 β-propeller domains 3-4), we found that treatment with SOSTDC1 promotes signaling. It is hypothesized that this may be due to a SOSTDC1 pre-formed LRP6 dimer complex on the cell surface. The determination of the SOSTDC1 crystal structure unveils a new dimerization motif among the DAN family and allows for a better understanding of the mechanism of action of SOSTDC1. A molecular understanding of SOSTDC1 will support characterization of the Wnt-BMP axis in development.

